# Right Valsalva Sinus Aneurysm Protruding Into the Right Ventricle: A Case Report

**DOI:** 10.15171/jcvtr.2015.27

**Published:** 2015

**Authors:** Ata H. Afshar, Sergei Kolesnikov, Leili Pourafkari, Nader D. Nader

**Affiliations:** ^1^ Department of Anesthesiology, State University of New York at Buffalo, Buffalo, NY, USA; ^2^ Cardiovascular Research Center, Tabriz University of Medical Sciences, Tabriz, Iran

**Keywords:** Aortic Aneurysm, Sinus of Valsalva, Rupture of Aortic Aneurysm

## Abstract

A separation between the aortic media and annulus fibrosus causes a rare cardiac abnormality called sinus of Valsalva aneurysm (SVA) that may be congenital or acquired. It is more prevalent in the right coronary sinus (65%-85%) but it has been seen rarely in non-coronary (10%-30%) or Left coronary sinus (<5%). The most common complication is rupture of the Aneurysm. We present an 80-year-old male with expanding right Valsalva sinus aneurysm and protruding into right ventricle. The conventional treatment is surgical repair under cardio-pulmonary bypass or percutaneous catheter closure. The aneurysm was successfully excised surgically under direct guidance of trans-esophageal echocardiography (TEE).

## Case Presentation

**Figure 1:**
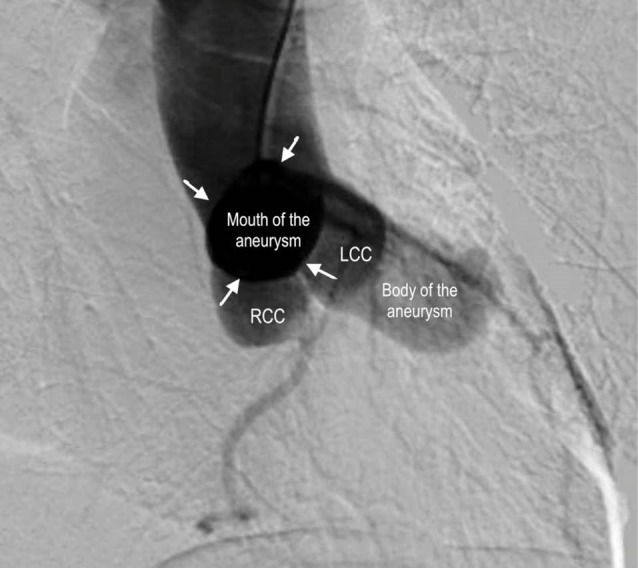
Aortogram in RAO projection showing the large aneurysm of the right valsalva sinus.

Eighty-year-old man with past medical history of hypertension, type 2 diabetes, dyslipidemia was diagnosed with an aneurysm of the Valsalva sinus in 2008. His last computerized tomographic angiography in early 2014 showed an enlargement of the aneurysm from 2.1 cm to 3.3 cm. Patient remained asymptomatic. Physical exam was benign except for a grade III/VI mid-systolic ejection murmur over the 5th intercostal space on left mid-clavicular line. Aortography was done in the LAO and RAO projection. In the RAO projection, a large aneurysm of the right sinus was seen protruding well beyond the outline of the aorta and this did not seem to communicate with any other cardiac structures. There was no aortic insufficiency (Figure 1 and Supplementary 1). Coronary angiography showed normal epicardial coronary arteries. Left ventricular systolic function was normal with an estimated ejection fraction of 60%.

**Figure 2:**
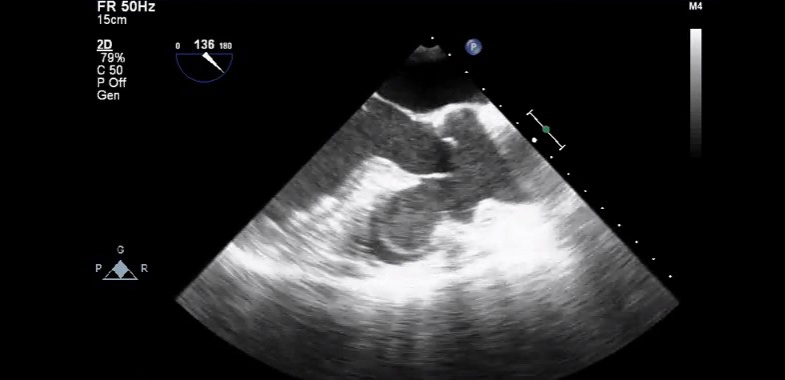
Two-dimentinal transesophageal echocardiographic midesophageal long-axis view showing the aneurysm of right valsalva sinus

**Figure 3:**
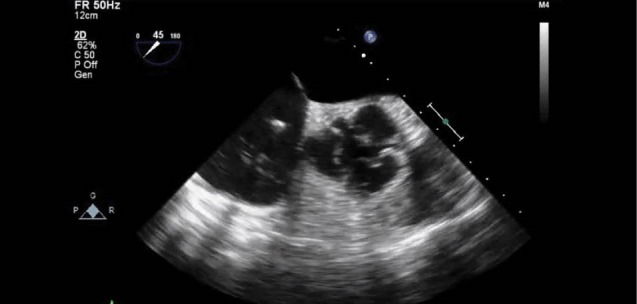
Post-operative transesophageal echocardiographic image through short axis of aortic valve showing the result of repair. There was no aortic insufficiency.

The surgery was performed through a median sternotomy. Intra-operative trans-esophageal echocardiography (TEE) was performed to monitor the procedure. Pre op TEE views are shown (Figure 2) The aorta was cross-clamped and cold potassium blood cardioplegia was given in an antegrade and continuous retrograde direction. An ascending aortotomy was performed. There was an excellent rim of aortic tissue completely surrounding the aneurysm neck. The right coronary artery was completely freed. A bovine pericardial patch was fashioned. The aortic valve appeared to be untouched and competent. The patient was then weaned off from cardiopulmonary bypass without any need for inotropic support. Post-Op TEE views are shown (Figure 3). Aortic valve regurgitation or residual shunt was excluded.

The patient remained stable and returned to the cardiac intensive care unit (CVICU). He was completely paced upon CVICU admission. His intrinsic rhythm was normal sinus rhythm. In postoperative day 3, he developed paroxysmal atrial fibrillation with a rapid ventricular response. His rhythm returned to normal sinus rhythm with intravenous amiodarone. He continued to remain in normal sinus rhythm during the following days. He was converted to oral amiodarone and discharged home on 7th postoperative day.

## Discussion

Sinus of Valsalva aneurysm (SVA) refers to the separation between the aortic media and annulus fibrosus of that may be congenital or acquired. It is more prevalent in the right coronary sinus (65%-85%) but it has been seen rarely in non-coronary (10%-30%) or left coronary sinus (<5%).^1^ The most common complication is rupture of the aneurysm. It can cause left to right shunt if ruptured into right side of the heart or could cause aorto-cardiac shunt and acute progressive heart failure.^2^ About 30% to 40% of patients may have coexisting cardiac abnormalities most commonly a ventricular septal defect or aortic regurgitation.^3^ Most of the patients become symptomatic between 30 and 45 years of age. Rupture of the aneurysm could lead to acute heart failure presenting with acute onset of shortness of breath, chest pain and fatigue.^4^ Most Valsalva aneurysms protrude into right cardiac chambers but protrusion and rupture into pericardium, left cardiac chambers and pulmonary artery have also been reported.^5^ TEE has been very helpful for diagnosing ruptured and intact aneurysm and has also proved to be a great guidance tool for intra operative repair. It provides information regarding involved sinuses, protrusion, and associated shunt or coexisting cardiac abnormalities. Cardiac angiography is also performed to evaluate coronary perfusion prior to surgery. Magnetic resonance imaging might be useful in diagnosing the coexisting cardiac lesions more precisely. Non-symptomatic and intact aneurysms are managed conservatively. Surgical resection is generally recommended in symptomatic cases, when there is compression or distortion of surrounding structures, in cases of rupture, and in the cases in which the SVA is incidentally discovered at the time of surgery for other cardiac reasons.^6^ Generally close follow-up is advised for unruptured SVA aneurysms in the absence of coexisting cardiac pathology.^7^ While specific guidelines for repair of a SVA has not been confirmed, it is generally accepted to follow the guidelines for aortic root aneurysm size to decide for surgical intervention.^8^

Currently, the optimal care for a ruptured sinus of Valsalva aneurysm is surgical repair, however, transthoracic minimally invasive closure has also recently been proposed.^8^ The first case of transcatheter closure of SVA using Rashkind umbrealla was reported in 1994.^9^ Since then multiple reports have described different approaches for percutaneous closure of SVA using septal occluder device, ductal occluder and Amplatzer vascular plug.^8^ Technique of “Dual Exposure” is the most used surgical technique to explore both aorta and the chamber of termination. The aneurismal sac would be excised and the defect will be sutured or patched. The occurrence of aortic regurgitation has been reported postoperatively.

## Conclusion

Echocardiography and magnetic resonance imaging are useful tools for diagnosis, conservative management and intra-operative guidance. Presence of a continuous murmur along with sudden onset of chest pain and shortness of breath might be secondary to a ruptured sinus of Valsalva. Surgical repair of the aneurysm still remains the standard treatment.

## Ethical Issues

The study was approval by local Ethics Committee..

## Competing Interests

None.

## Supplementary files

 Supplementary 1 consists of video file.
